# *Drosophila* EYA Regulates the Immune Response against DNA through an Evolutionarily Conserved Threonine Phosphatase Motif

**DOI:** 10.1371/journal.pone.0042725

**Published:** 2012-08-15

**Authors:** Xi Liu, Teruyuki Sano, Yongsheng Guan, Shigekazu Nagata, Jules A. Hoffmann, Hidehiro Fukuyama

**Affiliations:** 1 INSERM Equipe Avenir, CNRS UPR9022, Institut de Biologie Moléculaire et Cellulaire, Strasbourg, France; 2 University of Strasbourg, Strasbourg, France; 3 CNRS UPR9022, Institut de Biologie Moléculaire et Cellulaire, Strasbourg, France; 4 Department of Medical Chemistry, Graduate School of Medicine, Kyoto University, Kyoto, Japan; 5 Solution Oriented Research for Science and Technology, and Core Research for Evolutional Science and Technology, Japan Science and Technology Corporation, Kyoto, Japan; Indiana University, United States of America

## Abstract

Innate immune responses against DNA are essential to counter both pathogen infections and tissue damages. Mammalian EYAs were recently shown to play a role in regulating the innate immune responses against DNA. Here, we demonstrate that the unique *Drosophila eya* gene is also involved in the response specific to DNA. Haploinsufficiency of *eya* in mutants deficient for lysosomal DNase activity (DNaseII) reduces antimicrobial peptide gene expression, a hallmark for immune responses in flies. Like the mammalian orthologues, *Drosophila* EYA features a N-terminal threonine and C-terminal tyrosine phosphatase domain. Through the generation of a series of mutant EYA fly strains, we show that the threonine phosphatase domain, but not the tyrosine phosphatase domain, is responsible for the innate immune response against DNA. A similar role for the threonine phosphatase domain in mammalian EYA4 had been surmised on the basis of *in vitro* studies. Furthermore EYA associates with IKKβ and full-length RELISH, and the induction of the IMD pathway-dependent antimicrobial peptide gene is independent of SO. Our data provide the first *in vivo* demonstration for the immune function of EYA and point to their conserved immune function in response to endogenous DNA, throughout evolution.

## Introduction

In humans, innate inflammation is frequently linked to various types of diseases, namely to autoimmune diseases [1]. However, it is still unclear in many instances how inflammation is initiated and chronically maintained. Pathogen invasions and/or subsequent changes of cellular integrity can cause inflammation and DNA has been reported to be a strong immune stimulator under such conditions [2], [3]. How cells recognize, and respond to, DNA are among the challenging questions in the field of inflammation, particularly in humans.

Several DNA recognition molecules have been recently identified. Among these are TLR-9 [4], Absent in Melanoma 2 (AIM2) [5], [6], Interferon gamma-inducible protein 16 (IFI16) [7], DNA-dependent activator of IRFs (DAI) [8], High-mobility group box protein 1 (HMGBP1) [9], and Leucine-rich repeat flightless-interacting protein 1 (LRRFIP1) [10]. These identifications were routinely based on the analysis of synthetic DNA-mediated immune reactions [11], [12]. Recent studies have addressed *DNaseII* deficient animals or cells to further our understanding on the DNA sensing mechanisms [13] and on the downstream signaling events.

DNaseII is an evolutionarily conserved acid DNase localized in lysosomes [14]. Macrophages in the fetal liver and thymus of *DNaseII* ^−/−^ mice cannot digest DNA of engulfed dead cells, or DNA of nuclei expelled from erythroid precursors [15], [16]. This results in their production of pro-inflammatory cytokines such as interferon-β (IFN-β) and tumor necrosis factor-α (TNF-α) [17], [18]. As a result of excessive cytokine production, *DNaseII* ^−/−^ mice suffer from anemia during embryonic stages and later, as adults, from polyarthritis. Crosses of *DNaseII* ^−/−^ mice with mice deficient for various immune response-related signaling molecules conclusively showed that (1) the DNA-dependent IFN-β gene expression is TLR-independent and IRF-3/7-dependent and that (2) the DNA-dependent TNF-α gene expression is both independent of TLR- and IRF-3/7- signaling [19], [20].

To elucidate the molecular mechanisms of DNA sensing in *DNaseII* ^−/−^ mice, Okabe et al. recently performed expression cloning and reported that Eyes absent 4 (*EYA4*) enhances the innate immune responses against DNA by activating IRF3 and NF-κB [21]. EYA was originally identified in *Drosophila* as a transcription factor that is essential for eye development [22]. Mammals have four *EYA* paralogs, *EYA1-4*. Mutations in human *EYA1* cause branchio-oto-renal (BOR) syndrome, an autosomal dominant genetic disorder, affecting necks, ears, and kidneys [23], [24], [25]. *EYA1^−/−^* mice also show renal abnormalities and a conductive hearing loss similar to BOR syndrome [26]. *EYA3^−/−^* mice have a minor defect in locomotion with some deficits of respiratory, muscle and heart functions [27], and *EYA4^−/−^* mice show a defect in eustachian tube and middle ear [28]. *EYA2^−/−^* mice have not yet been generated. Several groups recently showed that EYA proteins carry C-terminally an evolutionarily conserved domain with tyrosine phosphatase activity [29], [30]. The detailed biochemical analysis by Okabe et al. [21]. Further identified a N-terminal threonine phosphatase domain in mouse EYA4, and showed that this domain is responsible for the innate immune responses observed against DNA.

*Drosophila* has a single orthologue of mammalian *DNaseII*. Interestingly, Mukae et al. found that flies carrying a hypomorphic mutation in the *DNaseII* gene (*DNaseII^lo^*) constitutively express the anti-bacterial peptides Attacin A and Diptericin, but not the anti-fungal peptide Drosomycin [31]. Transcription of antimicrobial peptides in *Drosophila* is essentially under the control of two regulatory pathways: the IMD pathway (largely similar to the TNF/TNF-R pathway in mammals) controls the transcriptions of *Attacin A* and *Diptericin*, whereas the TOLL pathway regulates the transcription of *Drosomycin* [32]. The observations by Mukae et al. therefore suggest that excess DNA in the *DNaseII* deficient flies could activate the IMD pathway, but not the TOLL pathway.

Induction of immune response to endogenous DNA had not been established before the study of Mukae et al. in 2002 [31] and few studies have been devoted to this aspect of the innate immune response since. We decided to utilize *Drosophila DNaseII* deficient flies as a model to study the immune response against DNA. By taking *Attacin A* induction as a read-out, we first have addressed here the function of the *eya* gene of *Drosophila*. The fact that *Drosophila* has a single *eya* gene, would allow for precise *in vivo* analysis of the function of this gene in the immune response. We now show that EYA indeed plays a significant role in the expression of the IMD pathway-dependent antimicrobial peptide *Attacin A* in *DNaseII*-deficient flies. Interestingly, EYA is not involved in this *Attacin A* induction by Gram-negative bacteria as most potent stimulus in this pathway activation. In addition to the tyrosine-phosphatase activity, *Drosophila* EYA also carries a threonine-phosphatase activity at its N-terminus, as its mammalian counterparts. We demonstrate that this threonine phosphatase domain of *Drosophila* EYA is responsible for the innate immune responses against DNA in the *DNaseII* deficient model. In contrast, our results show that the tyrosine phosphatase domain governs eye development. We further reveal that EYA is associated with IKKβ and RELISH and AttacinA induction is independent of SO. Taken together with the Mukae et al and the Okabe et al studies in mice, our results point to a striking conservation of innate immune responses against endogenous DNA between *Drosophila* and mammals.

## Results

### *DNaseII* deficiency-dependent expression of *Attacin A* in *eya* mutant background

**Figure 1:**
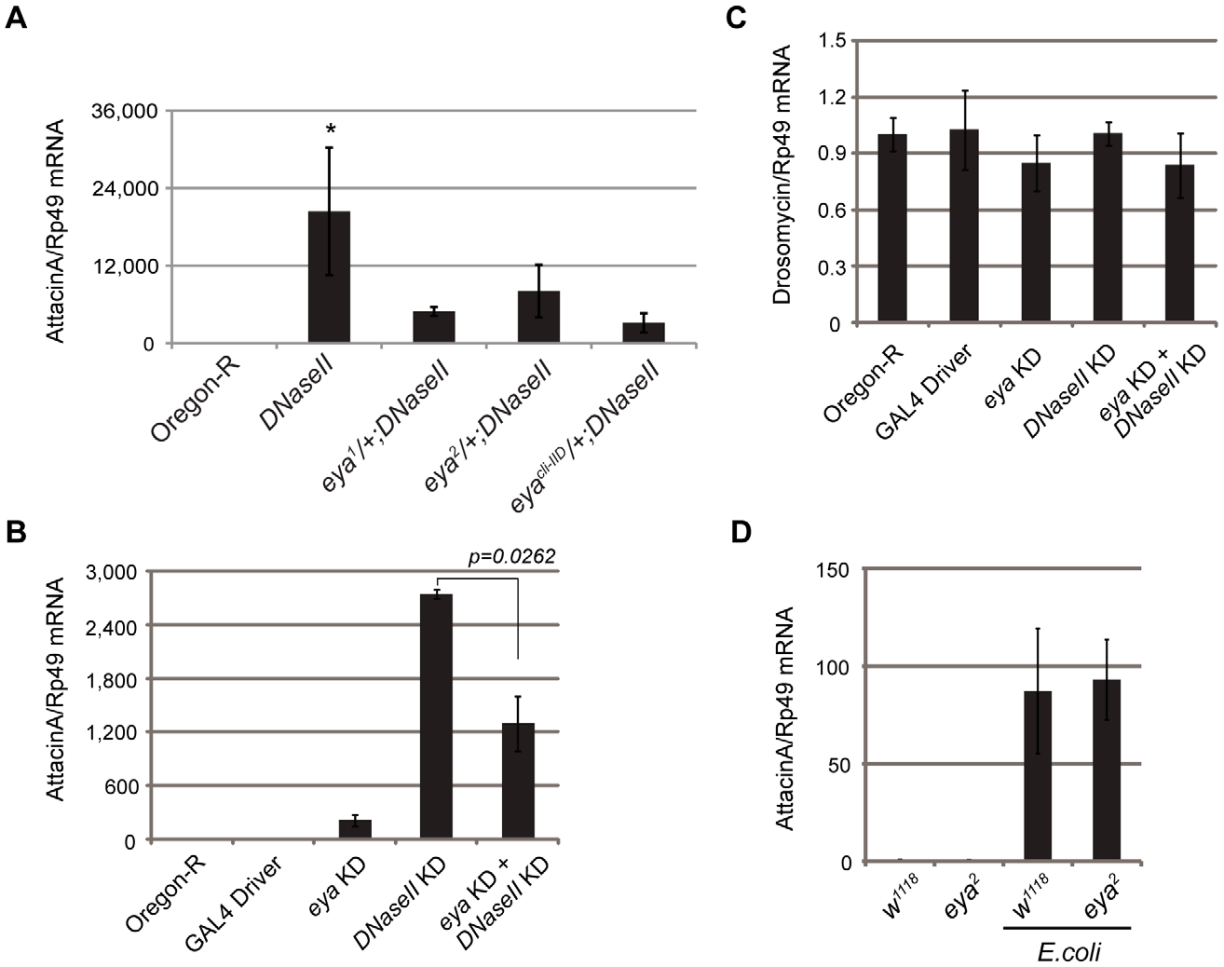
EYA is involved in innate immune responses against DNA. mRNA levels were determined for antimicrobial peptide genes by quantitative RT-PCR and normalized to Rp49 expression(also known as RpL32). The relative values are indicated against Oregon-R as wild-type control. (**A**) *Attacin A* expression for *DNaseII*, *eya* ^1^/+;*DNaseII*, *eya* ^2^/+;*DNaseII*, and *eya^cli-IID^*/+;*DNaseII* mutant flies. One-way ANOVA was performed and followed by Dunnett's multiple comparison test. * indicates statistically significance (p<0.0001) by comparing to Oregon-R. (**B**) *Attacin A* expression for single *eya*, single *DNaseII*, and double *DNaseII* and *eya* knockdown flies at 8 days after heat-shock treatment. dsRNAs were expressed using the GAL4-UAS system. The GAL4 driver line alone was included. Two-tailed unpaired Student's t-test was performed for statistic analysis. (**C**) *Drosomycin* expression using the same sets of flies as for (B). (**D**) *Attacin A* expression by *E.coli* challenge for *w^1118^* as wild-type control and *eya^2^* mutant flies. The value represents the average and standard deviation of 3–6 independent experiments. A pool of 5–20 adult flies per genotype was collected for each experiment.

We have first made use of three well-studied alleles of *eya*, which carry a 1.5 kb (*eya^1^*) or a 322 bp (*eya^2^*) deletion upstream of the transcription start site, and a nonsense loss-of-function mutation (*eya^cli-IID^*) that causes truncation of the EYA protein at amino acid 335 [33]. *Eya^1^* or *eya^2^* flies develop to adults deprived of eyes. *Eya^cli-IID^* flies are lethal at the embryonic stage. The *DNaseII^lo^* allele has a missense G-to-A mutation at nucleotide 668, converting a Serine residue to an Aspargine residue at amino acid position 223, which results in a reduction of the acid DNase activity [33]. *DNaseII* mutants develop normally to the adult stage. Interestingly, these flies constitutively express the antimicrobial peptide Attacin A [31]. We generated *eya^1^;DNaseII^lo^*, *eya^2^;DNaseII^lo^*, and *eya^cli-IID^;DNaseII^lo^* double mutant flies and monitored *Attacin A* expression. We noted that flies carrying homozygous *eya* and *DNaseII^lo^* double mutations died at the pupal stage and we therefore used adult *eya*/+;*DNaseII^lo^* flies to evaluate the effect of the *eya* gene in this context. As shown in Fig. 1A, *DNaseII* mutant flies constitutively expressed *Attacin A*, as previously reported [31]. In contrast, a heterozygous *eya^1^* mutation combined with *DNaseII* deficiency significantly reduced *Attacin A* mRNA levels. Similar results were obtained with the *eya^2^* and *eya^cli-IID^* alleles (Fig. 1A). To exclude possible effects of genetic background and/or aberrant development due to the gene deficiency/insufficiency, we generated an inducible *eya/DNaseII* knockdown system by RNA interference. In these experiments, we used established fly lines carrying dsRNAs for *eya* or/and *DNaseII* under a UAS promoter [34] and crossed them with a *hsp*-GAL4/*tub*-Gal80^ts^ fly line [35]. After heat shock treatment, we measured the *Attacin A* mRNA levels on day 8. As shown in Fig. 1B, heat shock itself did not induce the *Attacin A* gene in the wild-type and GAL4 driver lines. The knockdown of the *DNaseII* gene, but not that of the *eya* gene, strongly activated *Attacin A*: Interestingly the knockdown of *eya* resulted in a strong reduction of the *DNaseII* knockdown-induced *Attacin A* expression. We observed a very low level of expression of *Drosomycin* in the Oregon-R strain, and this expression remained unchanged in knockdowns of *eya* and/or of *DNaseII* (Fig. 1C). These results provide an *in vivo* demonstration that *Drosophila eya* is involved in the immune response to DNA. *Attacin A* can be induced by Gram-negative bacterial challenge through the *Drosophila* IMD pathway. We investigated the role of EYA in the immune response during bacterial infection. As Fig. 1D shows, the *Attacin A* mRNA level was increased by bacterial challenge. However, its level was not altered in *eya^2^* mutants. This result indicates that EYA plays a role in the immune response specifically against DNA. It is important to note that the genetic background affects the basal mRNA levels of *Attacin A*. However, Attacin A induction by *DNaseII* deficiency is consistent in both cases, mutant and RNAi transgenic flies. Two additional observations further support this conclusion: (1) *cn bw*-background *DNaseII* mutants established by backcrossing also show constitutive expression of Attacin A and (2) the rescue experiment showed that the Attacin A induction is dependent on *DNaseII* deficiency (X.L. and H.F., unpublished results).

### *In vitro* functional analysis of the threonine phosphatase domain in recombinant *Drosophila* EYA mutant proteins

As mentioned in the Introduction, the four mammalian EYAs carry a C-terminal tyrosine phosphatase domain, also called EYA domain [36], which is conserved in flies. In addition, the mammalian and fly EYA proteins have a well-conserved N terminal threonine phosphatase domain with six tyrosine residues [21].

**Figure 2:**
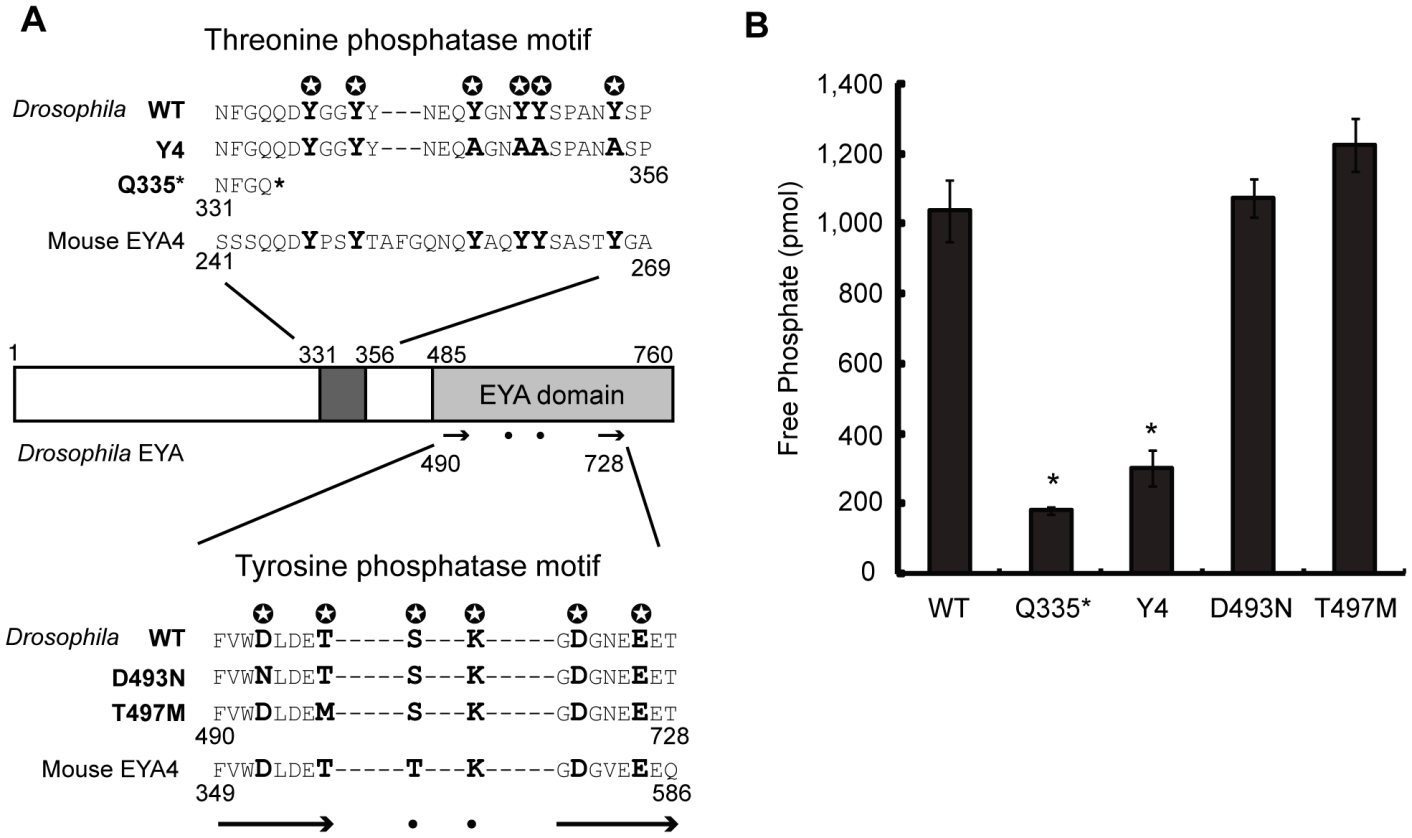
Threonine phosphatase domain in recombinant *Drosophila* EYA mutant proteins. (**A**) Schematic view of *Drosophila* EYA with various mutant forms, WT, Y4, Q355*, D493N, and T497M. Two distinct phosphatase motifs are shown. Numbers indicate the positions of amino acid residues. 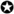 and bold letter indicate evolutionarily conserved amino acids in the motifs. Arrows and dots in the EYA domain correspond to those shown in the magnified view with amino acid sequences. Two motifs in amino acid sequences of Mouse EYA4 are shown. (**B**) *Drosophila* EYA threonine phosphatase activities of WT, Q355*, Y4, D493N, T497M were measured. Free phosphate in mol is indicated. One-way ANOVA was performed and followed by Dunnett's multiple comparison test. * indicates statistically significance (p<0.001) by comparing to WT.

To characterize the *Drosophila* EYA threonine phosphatase activity in the present context, we expressed in mammalian 293 T cells a series of recombinant *Drosophila* EYA proteins: (1) Q335*, a nonsense mutation that causes truncation of the EYA protein at amino acid 335 and corresponds to the *eya^cli-IID^* loss-of-function allele [37] in the literature; this protein carries neither the tyrosine nor the threonine phosphatase motifs; (2) T497M, a missense mutation of the EYA domain (Fig. 2A), known as the *eya^E11^* allele [37]; (3) D497N; this allele was experimentally generated for recombinant protein EYA4 by a D493N replacement, which abolishes the tyrosine phosphatase activity [21], [29], [30]; (4) Y4 is an experimentally introduced allele, in which four tyrosine residues have been replaced in the threonine phosphatase domain, resulting in the total loss of the threonine phosphatase activity [21]; (5) WT refers to the wild-type EYA. After expression in 293 T cells, the recombinant proteins were purified with anti-Flag mAb and served to determine *in vitro* threonine-phosphatase activities using a phospho-threonine peptide. The results were as follows (Fig. 2B): The threonine-phosphatase activities of D493N and T497M were similar to that of WT. In contrast the threonine-phosphatase activities of the Y4 and that of Q335* were severely diminished. These results demonstrate that *Drosophila* EYA has a threonine phosphatase activity governed by the six-tyrosine-residues motif, and that this motif is not only evolutionarily but also functionally conserved from insects to mammals.

### *In vivo* analysis of the role of the EYA threonine-phosphatase domain in *Attacin A* expression

**Figure 3:**
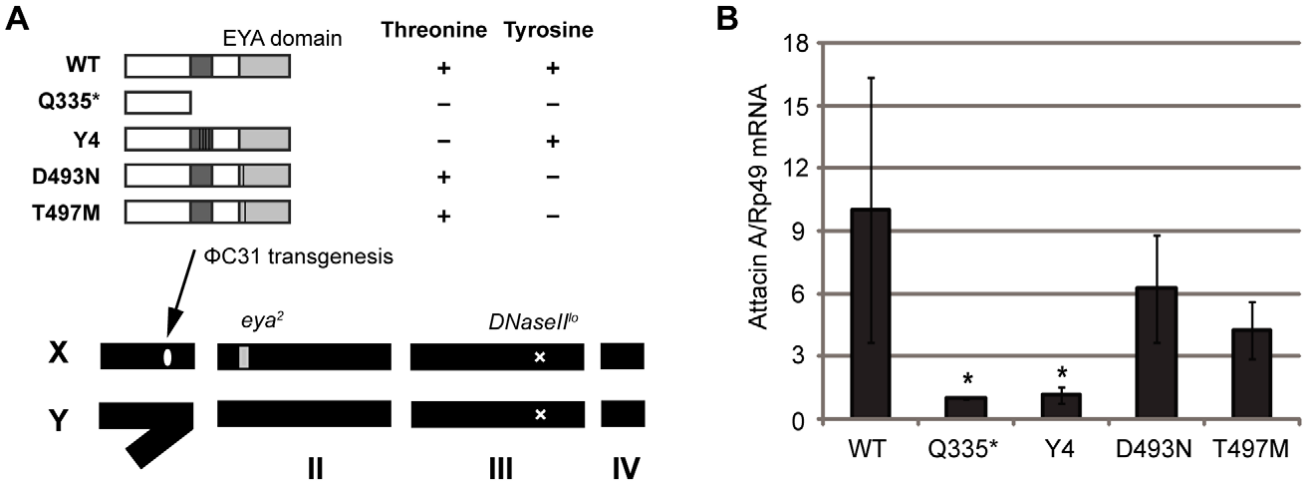
*In vivo* analysis of the role of the EYA threonine-phosphatase domain in *Attacin A* expression. (**A**) Schematic views of *eya* rescue transgenic fly lines. Various EYA transgenes were introduced on the X chromosome by ΦC31 transgenesis. These flies carry *eya^2^* heterozygous and *DNaseII^lo^* homozygous alleles on the 2^nd^ and the 3^rd^ chromosomes, respectively. The presence or absence of threonine and tyrosine phosphatase motifs in each transgene is shown by + or −. Bold lines in the transgenes are locations of replacement of amino acids. (**B**) *Attacin A* mRNA level was measured by quantitative RT-PCR and normalized to Rp49 expression(also known as RpL32). The relative values are indicated against Q355*. The value represents the average and standard deviation of three independent experiments. A pool of 5–7 adult flies per genotype was collected in each experiment. One-way ANOVA was performed (p = 0.0261) and followed by Dunnett's multiple comparison test. * indicates statistically significance (p<0.05) by comparing to WT.

With this information at hand, we next examined which of the threonine versus tyrosine phosphatase domains of *Drosophila* EYA is responsible for the innate immune responses against DNA. We generated, through bacteriophage ΦC31 integrase-mediated transgenesis [38], [39], fly lines expressing the various alleles of *Drosophila* EYA described above, i.e., WT, Q335*, T497M, D493N, and Y4. Expression was driven by a heat-shock promoter [22]. In brief, this transgenesis technique integrates a transgene (in our case, various forms of *eya* cDNAs) at the same specific site in the genome, allowing to make quantitative comparisons between different forms of EYA. We then crossed these *eya* transgenic flies to the homozygous *eya^2^*;*DNaseII^lo^* strains. As mentioned above, homozygous *eya^2^;DNaseII^lo^* flies show nearly full lethality; in the mutant series we observed some degree of rescue of the lethality with the WT *eya* transgenes, but barely with the other transgenes. We therefore decided to continue our experiments in an *eya^2^* heterozygous and *DNaseII^lo^* homozygous background. Under these conditions most of the flies survived. In WT flies, we observed a high level of *Attacin A* induction. When we compared this level to the transgenic line carrying the dual phosphatase inactive form, Q335*, we noted a dramatically lowered level of expression. Flies carrying a transgene in which only the tyrosine phosphatase function was lost (D493N and T497M) showed no significant difference with those of WT flies. Finally and most interestingly in the present context, flies carrying the Y4 construct, i.e. with an inactive threonine phosphatase domain and a wild type tyrosine phosphatase domain, showed the same level of expression as the forms with dual inactive phosphatase domains. Since the different effects of *eya* transgenes on the *Attacin A* expression could not be explained by the expression levels of the transgenes (Fig. S1, the same set of RNA in Fig. 3B was used), our results clearly point to the threonine phosphatase domain as the domain up-regulating the level of expression of *Attacin A* induced in the *DNaseII* deficient background.

### The threonine phosphatase domain is not required for eye development

**Figure 4:**
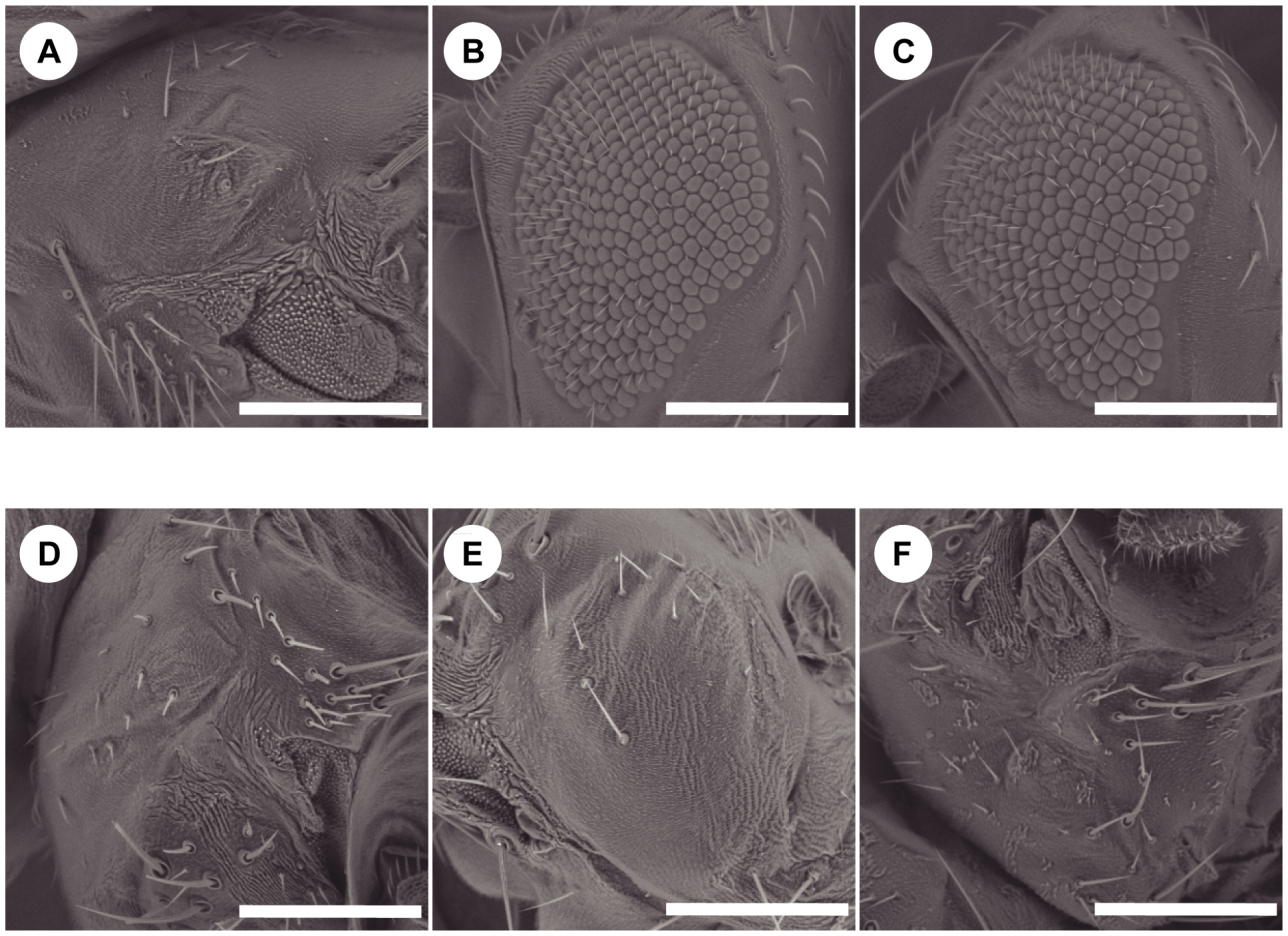
Delineation of phosphatase activities for eye development. Scanning electron micrographs of adult eyes. (A) *eya* ^2^, (B) WT;*eya* ^2^, (C) Y4;*eya* ^2^, (D) Q355*;*eya* ^2^, (E) D493N;*eya* ^2^ (F) T497M;*eya* ^2^ Scale bars correspond to 300 µm in length.

We next investigated the impact of the two different phosphatase activities of EYA on eye development. Although previous studies indicated that the tyrosine-phosphatase activity is required for eye development [29], [40], the possibility that the threonine phosphatase activity also affects eye development could not be excluded. As shown in Fig. 4, the eyes did not develop in flies carrying the *eya^2^* mutation, as reported previously [22]. The inducible expression of the WT and Y4 forms of EYA at the stages from egg to pupae rescued the *eyes absent* phenotype. In contrast, the expression of the EYA forms of Q355*, D493N, and T497M could not rescue the phenotype. These results indicate that the tyrosine phosphatase activity of EYA is required for eye development, but that its threonine-phosphatase is dispensable.

### EYA associates with IKKβ and RELISH and *AttacinA* induction is independent of transcription factor SO

**Figure 5:**
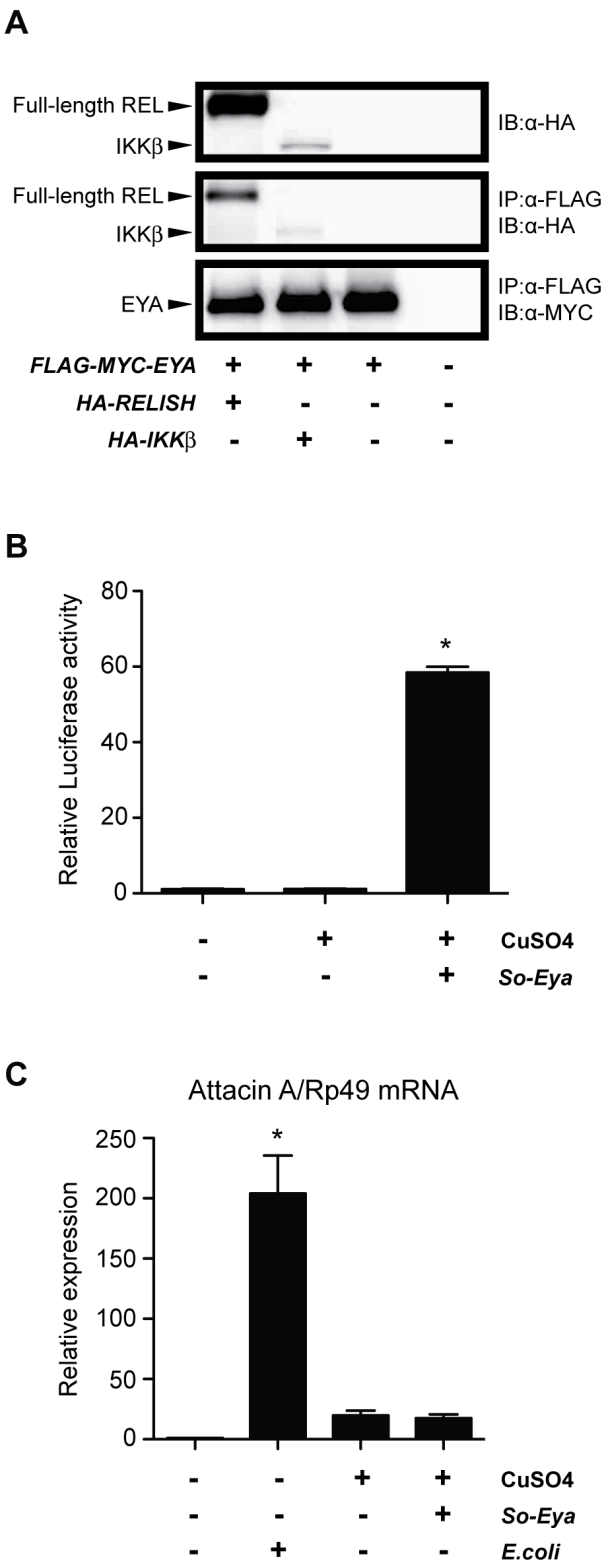
The link between EYA and the IMD pathway. (**A**) Association studies between EYA and IKKβ or RELISH. FLAG-MYC-EYA was co-expressed with HA-IKKβ or HA-RELISH in S2 cells and immunoprecipitated (IP) with anti-FLAG antibody followed by immunobloting (IB). IKKβ and RELISH, or EYA were detected by anti-HA antibody or anti-MYC antibody, respectively. Five percent of total cell lysates as the input was shown on the top panel. (**B**) So-Eya co-expression induced the reporter activity. Luciferase activities of the reporter ARE-luciferase were measured and normalized to β-gal activities of Act5C-lacZ. The relative values against control of non-transfected and non-induced cells were shown. The value represents the average and standard deviation of 3–4 independent experiments. (**C**) Attacin A mRNA level was determined quantitative RT-PCR and normalized to Rp49 expression using the same sets in (B) except stimulated experimental set: S2 cells were stimulated by heat-killed *E.coli* (DH5α, heat-treated at 60°C for 1 h) for 16 h at 1∶20 MOI. The relative values against control of non-transfected, non-induced, and non-stimulated cells were shown. The value represents the average and standard deviation of 3–4 independent experiments. One-way ANOVA was performed (p<0.0001) and followed by Dunnett's multiple comparison test. * indicates statistically significance (p<0.0001) by comparing to control.

To obtain the mechanistic view of a link between EYA and the IMD pathway, we performed protein-protein association studies. Since the threonine phosphatase activity of EYA plays an important role for immune responses in *DNaseII* deficiency model, we reasoned to investigate two potential phospho-substrates of the IMD pathway: IKKβ and RELISH [41], [42]. When FLAG-MYC-tagged EYA and hemagglutinin (HA)-tagged IKKβ or RELISH were expressed in S2 cells, IKKβ and RELISH associated with EYA (Fig. 5A).

On the other hand, EYA binds to the homeobox transcription factor Sine Oculis (SO) [43]. To determine whether the *AttacinA* can be a target of the So-Eya complex, we co-transfected *eya* and *so* in S2 cells with reporter plasmid ARE-luciferase [44]. As previously reported [44], [45], co-transfection of *eya* and *so* activated ARE-luciferase 50-fold over the reporter alone (Fig. 5B). In this condition, *AttacinA* induction was not observed while heat-killed *E.coli* challenge induced *AttacinA* by more than 200-fold (Fig. 5C). These results indicate that *AttacinA* is not a target of the complex of EYA and SO.

## Discussion

We are interested in understanding how endogenous ligands can induce immune responses in *Drosophila* and whether the receptors and downstream signaling cascades are similar to those which are activated upon well-defined microbial stimuli (bacteria, fungi, virus) [46], [47]. The discoveries that mice and flies deficient for lysosomal DNase activities mount an immune response, as evidenced by the constitutive expression of IFN-β or of the antibacterial peptide Attacin A respectively, was of great interest in this context. The recent report by Okabe et al. that this innate response to undigested DNA is regulated in mice by the *eya* gene(initially discovered in *Drosophila* eye development) stimulated our interest in the potential role of this gene in the immune response of *Drosophila*. We provide four essential findings: (1) the immune response induced by undigested DNA in *DNaseII* deficient flies requires the *eya* gene; (2) the N-terminal threonine phosphatase domain of the EYA protein is responsible for this function, whereas that of the C-terminal tyrosine-phosphatase domain is dispensable; (3) EYA associates with IKKβ and RELISH and the So-Eya complex does not induce *Attacin A*; (4) the role of EYA proteins is conserved in this specific immune context between flies and mammals. We present the first *in vivo* demonstration for the role of the threonine-phosphatase domain of EYA proteins, which were so far surmised only on the basis of the *in vitro* studies in mice.

The two main questions, unanswered to date, pertain to (1) the identity of the DNA sensor (receptor) in flies; (2) the target molecule for the threonine-phosphatase activity of EYA.

As regards the first question, we have no firm data regarding DNA sensors in flies at present. In mammals, several molecules, play more or less well defined roles in DNA recognition, namely TLR9, AIM2, DAI, for which there are no homologues in flies. Identifying the DNA sensor in *Drosophila* is clearly a priority in the field. Of note, the sensor for DNA that accumulates in macrophages in *DNaseII* deficient mice has not yet been firmly identified. TLR9, which would appear as a good candidate, is not involved, as in *DNaseII^−/−^ TLR9^−/−^* mice the innate immune response to accumulated DNA is unaffected.

How does EYA activate the IMD pathway to control expression of the *Attacin A* gene? Our transactivation and transcription assay indicates that *AttacinA* induction is not regulated by the So-Eya complex. Furthermore our protein-protein association studies suggest the link between EYA and the IMD pathway at the level of IKKβ and RELISH. It is noteworthy that EYA can associate with full-length RELISH. In general, the full-length RELISH is located in the cytoplasm and activated via two events: (1) phosphorylation by IKKβ and (2) cleavage by DREDD (similar to Caspase8/10), and eventually truncated N-terminal half of RELISH translocates to the nucleus to regulate transcriptions of target genes such as antimicrobial peptide genes [41], [48]. We propose that EYA can make a complex with IKKβ and RELISH in the cytoplasm, and activate the IMD pathway at this level. Interestingly, *Eya^2^* mutants can respond to Gram-negative bacteria. These observations clearly exclude possibilities that EYA directly involves the phosphorylation of Serine 528 and Serine 529 of RELISH, which are targets of IKKβ and required for target gene expression. It is interesting to investigate the role of EYA on the phosphorylation status of Serines/Threonines in 107-aa C-terminal region in RELISH, which is also targets of IKKβ and required for the interaction between RELISH and IKKβ [41]. Of interest in the present context is the observation that both *Drosophila* and mammalian EYAs have two MAPK phosphorylation sites and that the *Drosophila* ERK and p38 MAPKs can phosphorylate *Drosophila* EYA *in vitro* [49]. Recently Morilo *et al.* demonstrated that NMO phosphorylates EYA and potentiates the transactivation function to enhance transcription of So-Eya target genes during eye specification and development [50]. Further, we now know that recombinant mouse EYA4 proteins produced in 293 T cells are phosphorylated (T.S., and S.N., unpublished results). The precise mechanism of dephosphorylation of target protein by EYA needs to be elucidated [51], [52].

In the mammalian system, EYA4 has been reported to be recruited by the dsRNA homologue poly (I:C) to the IPS-1 complex to activate the IRF3 and NF-κB pathways. This complex consists of various regulating molecules, namely RIG-I, STING, and NLRX1 [21]. No clear-cut homologue of any of these molecules were found in *Drosophila* till now (although RIG-I and *Drosophila* Dicer-2 share a helicase domain with the significant amino acid sequence similarity [53]). The components of the complex responsible for DNA-sensing remain to be revealed. EYA is the first identified molecule that is found in both insects and mammals in DNA sensing cascade.

Lastly, a surprising result of this study was that while both *DNaseII*- and *eya^1^- or eya^2^*- deficient flies develop normally, combining these two deficiencies is lethal at the pupal stage. The elucidation of this developmental arrest in *eya^1^;DNaseII^lo^* or *eya^2^;DNaseII^lo^* flies will hopefully shed light on DNA sensing and signal transduction in flies.

We anticipate that comparative studies on DNA sensing using *Drosophila* and mouse *DNaseII* deficient animals will facilitate the understanding of the molecular mechanisms of DNA-triggered innate inflammation.

## Materials and Methods

### Fly strains and maintenance

All flies were maintained with standard corn meal and yeast extract medium at 25°C at a light cycle- and humidity controlled-room. Oregon-R and *w^1118^* were used as wild type controls. Flies used for AMP expression experiment were raised in antibiotics cocktail medium (100 µg/ml ampicillin, 50 µg/ml vancomycin, 100 µg/ml neomycin and 100 µg/ml metronidazole) [54] to reduce the risk of contaminating bacteria infection that could induce antimicrobial peptide gene expression [47] (Fig. S2). *DNaseII*[lo] (FBal0002709), In(2L)*eya*, *eya*[1] (FBst0003631) and *eya*[2](FBal0030759) mutant flies were obtained from the Bloomington stock center. *Eya*[cli-IID] (FBal0001705) mutant flies were gifted from Dr.Nancy N. BONINI. RNAi transgenic for *DNaseII* (NIG#7780R-3) and for *eya* (FBst0465312) were purchased from the Fly stocks of National Institute of Genetics, Japan and the Vienna *Drosophila* RNAi Center, Austria, respectively. Inducible ubiquitous driver, *hsp*-GAL4/*tub*-GAL80^ts^ recombined fly line, was established by Dr.Ferrandon and described previously [35]. Heat shock-mediated GAL4 induction was performed as follows: all crossings with the *hsp*-GAL4/tub-GAL80^ts^ driver were made at 18°C, three days old hatched flies were inoculated at 29°C for 2 days, at 37°C for 30 minutes, at 18°C for 30 minutes, at 37°C for 30 minutes, and then flies were kept at 29°C. Note that the incubation at 37°C was processed in the water bath. For ΦC31-mediated transgenesis previously described [38], [39], we utilized X-linked attB landing fly line VK6-ΦC31 (y[1] w[1118] PBac{yellow[+]-attP-9A}VK00006; +; +; M{eGFP.vas-int.Dm}ZH-102D) gifted from Dr. Koen VENKEN. By crossings, we generated and analyzed on series of transgenic rescue lines, e.g. *hsp*-EYA/Y; *eya*[2]/*CyO*;*DNaseII*[lo]/*DNaseII*[lo] (Fig. 3A).

### Plasmids and Antibodies

The cDNA of *eya* was amplified from FBcl0108545 (*Drosophila* Genomics Resource Center) by PCR, cloned into pCR®8/GW/TOPO® TA cloning vector (Invitogen). The cDNAs of *Ikkb* (also called as *ird5*) and *Rel* were amplified using a cDNA library from S2 cells by PCR and cloned into pDONR207 vector (Invitogen). These pENTRY clones were verified by DNA sequencing and used for further plasmid constructions. Series of mutations were introduced by QuikChange® Lightning Site-Directed Mutagenesis Kit (Agilent Technologies) and the sequences were confirmed by DNA sequencing. These mutations includes: Y4 [21], D493N [21], T497M [37], and Q335* [37]. Primers used in this report are listed in Table S1. Based on pCaSpeR-hs (FBmc0000179) vector, the Gateway®-based destination vector pCaSpeR-attB-hsp-FW was generated for transgenesis. This vector containing the attB site for ΦC31-mediated transgenesis, Gateway® cassette fused with three repeated Flag sequence obtained from pTFW vector (the *Drosophila* Genomics Resource Center) for amino-terminal tagging. After LR reactions between pCaSpeR-attB-hsp-FW and pCR®8-N-dEYA (WT, Y4, D493N, T497M, and Q335*), series of pCaSpeR-attB-hsp-dEYA vectors were generated, followed by transgenesis using X-linked VK6-ΦC31. For the production of recombinant *Drosophila* EYAs, series of *eya* cDNAs in pCR®8/GW/TOPO® were transferred to mammalian expression vector pEF-BOS [55]. For biochemical analysis, Gateway®^−^based destination vector pMT-HW was generated based on pHHW (the *Drosophila* Genomics Resource Center) and pMT/V5-His-A vector (Invitrogen). The vector contains Metallothionein-inducible promoter and hemagglutinin (HA) tag at amino-terminal. pAFMW destination vector contains Actin 5C promoter and dual tags of FLAG and MYC at amino-terminal. By LR reactions with these two destination vectors, we generated pMT-HA-IKKβ, pMT-HA-REL, and pAFMW-dEYA. For transactivation and transcription assay, pARE-luciferase, pRmHa3-Flag-SO and pRmHa3-Flag-EYA are kindly provided by Dr. Ilaria REBAY [44]. pACH110 containing Actin 5C promoter and LacZ gene was used for normalization of transfection efficiency. Anti-FLAG® M2 antibody (SIGMA-ALDRICH) for immunoprecipitation, Rabbit polyclonal anti-HA antibody (SIGMA-ALDRICH), anti-MYC antibody (BOEHRINGER INGELHEIM), and Horseradish Peroxidase (HRP)-conjugated anti-Rabbit or anti-mouse IgG antibody (SIGMA-ALDRICH) as secondary antibodies for immunoblotting were used in this study.

### Protein expression, purification, and thereonine phosphatase activity assay

The *Drosophila* EYA proteins were produced in 293 T cells and purified using anti-Flag M2 affinity gel (SIGMA-ALDRICH) as described previously [21]. Briefly, series of pEF-BOS-dEYAs were transfected by Ca-Phosphate co-precipitation method, and cells were lysed and the supernatants after centrifugation was subjected to the purification. To quantify the phosphatase activity, the purified recombinant EYA was incubated with the phosphorylated synthetic peptide KR(pT)IRR at the concentration of 700 µM at 37°C for 60 min in 50 mM Tricine-KOH buffer (pH 8.0) containing 5 mM EDTA and 50 µM DTT using 0.5 pmol of a recombinant protein. The quantity of released phosphate was measured by colorimetric method using the malachite green-molybdate. The malachite green-ammonium molybdate phosphate complex was detected at 620 nm using a Micro Plate Reader (BioLumin 960).

### RNA analysis

Total RNA was extracted from adult flies using RNeasy Mini kit (Qiagen) or NucleoSpin® RNAII kit (Macherey-Nagel). TaqMan® RNA-to-CT™ 1-Step Kit (Applied Biosystems) was used for quantitative RT-PCR with TaqMan® Gene Expression Assays primers and probes (Applied Biosystems) using 7500 Fast Real-Time PCR System (Applied Biosystems). These assays include Attacin A (Dm02362218_s1), Drosomycin (Dm01822006_s1), and RpL32(also known as Rp49, Dm02151827_g1). A pool of 5–20 flies was collected for each experiment. The expressions of the antimicrobial peptide genes were normalized to the expression of the RpL32 gene for each sample. Each assay was performed in duplicated manner and the average of duplicates was used for a single experiment data. For gene expression of *eya* in flies and *AttacinA* in S2 cells, total RNA was extracted from adult flies or cells using NucleoSpin® RNAII kit (Macherey-Nagel) or TRI REAGENT® RT(Molecular Research Center). Reverse transcription was performed by RevertAid™ H Minus Reverse Transcriptase (Fermentas). Fast SYBR® Green Master Mix (Applied Biosystems) was used for Quantitative RT-PCR with 7500 Fast Real-Time PCR System (Applied Biosystems). *eya* or *AttacinA* mRNA levels were quantified and normalized to mRNA level of Rp49. Both primers (Q-*eya* or Q-*AttA* primers, and Q-*Rp49* primers) were listed in Table S1.

### Bacteria challenge

*Escherichia coli* (1106C) at 37°C shaker (INNOVA-44R) were inoculated for 7 to 8 hours until OD_600_ reached 0.6–0.8. The pellet was collected and washed twice with PBS. Finally *Escherichia coli* was suspended in PBS and OD_600_ was adjusted to 2.0 for injection. We injected 13.8 nl to each female fly by NANOJECT II (Drummond Scientific) with customized capillary needles (Drummond scientific) crafted by flaming/brown micropipette puller model P-97 (Sutter Instrument). Six hours after injection flies were collected for RNA extraction to determine AMP expression.

### Eye phenotype rescue experiment

Eggs were laid in culture media for 24 hours, and then processed the 1-hour heat shock at 37°C for every 8 hours using heat-blocks in the chamber until the white pupae stage. Hatched adult flies were counted for appearance of eye phenotype and frozen at −80°C. Frozen and dried flies were subjected to scanning electron microscope analysis using HITACHI TM-1000 Tabletop SEM (HITACHI).

### S2 cell transactivation and transcription assay

*Drosophila* S2 cells were transiently transfected by calcium phosphate method with 2.5 µg each of pRmHa3-Flag-SO and pRmHa3-Flag-EYA, 10 µg of the reporter plasmid ARE-luciferase, and 1 µg of pACH110 for normalizing transfection efficiency. After 16-hour incubation, cells were washed with PBS and CuSO_4_ was added to the culture medium at the final concentration of 1 mM for induction. Cells were harvested 24 h later and lysed in 70 µL reporter lysis buffer (Promega). Ten micro liter or 2.5 µL of lysates were used for luciferase assay or β-galactosidase assay, respectively. Both assays were performed in duplicate manner using Mithras LB940 multimode microplate reader (Berthold technologies). Three or four independent transfections were made for each experimental set.

### Immunoprecipitation and Immunoblotting

Six million of *Drosophila* S2 cells were transiently transfected with 5 µg each of pAFMW-dEYA and pMT-HA-IKKβ or pMT-HA-REL by calcium phosphate method. After 16-hour incubation, cells were washed with PBS and CuSO_4_ was added into the cell culture medium at the final concentration of 0.5 mM for induction. Cells were harvested 24 h later and then lysed with 200 µL of lysis buffer (50 mM Tris-HCl pH7.5, 10% Glycerol, 1% Triton X-100, 150 mM NaCl, 100 mM NaF, 5 µM ZnCl_2_, 1 mM Na_3_VO_4_, 10 mM EGTA pH8.0, 1 mM EDTA, protease inhibitor cocktails (ROCHE)) on ice for 30 min. After centrifugation, 10 µL of lysates were used for determining total protein expression level, and the rest was subjected to immunoprecipitation followed by immunoblotting. Anti-FLAG antibody was pre-incubated with 10 uL of Dynabeads® protein G (Invitrogen) in lysis buffer for 1 h at room tempreture followed by incubation with lysates for 2 h at 4°C. After washing 4 times by lysis buffer, the immunoprecipitates were boiled in 15 µL of 2× elution buffer (30% Glycerol, 0.15 M Tris-HCl PH6.8, 5% Sodium dodecyl sulfate, 0.02% Bromophenol blue, 0.72 M 2-Mercaptoethanol). For immunoblotting, proteins of immunoprecipitates and total lysates were resolved in Novex® 4–20% Tris-Glycine Gels (Invitrogen) followed by blotted to nylon membrane. Then the blot was incubated with anti-HA antibody(1∶1,000) or anti-Myc antibody(1∶1,000) followed by with Horseradish Peroxidase (HRP)-conjugated secondary antibodies(1∶15,000). The image was acquired by Fusion FX7 system (Vilber Lourmat) after incubated with Super Signal® West Dura substrates (Pierce). Three independent experiments were performed.

### Statistics

Two-tailed Student's t-test and one-way ANOVA were used for statistic analysis using Prism software.

## Supporting Information

Figure S1 **EYA expression in transgenic rescue fly lines.** The levels of *eya* mRNA were measured in rescue transgenic fly lines by quantitative RT-PCR and normalized to Rp49 mRNA levels. The relative expression values to Q355* are indicated. The value represents the average and standard deviation of three independent experiments. A pool of 5–7 adult flies per genotype was collected in each experiment.
(TIF)

Figure S2 **Bacteria growth in Antibiotics-treated flies.** Three flies grown in normal or antibiotics medium were squashed in 100 µl of PBS and then spread on LB plates. The LB plates were incubated at 25°C for two days.
(TIF)

Table S1 **Primers used in this study.**
(DOCX)
