# Supplementary figures and images for: Drosophila EYA Regulates the Immune Response against DNA through an Evolutionarily Conserved Threonine Phosphatase Motif
Source: PLoS One. 2012 Aug 15;7(8):e42725. doi: 10.1371/journal.pone.0042725 (PMC3419738; doi:10.1371/journal.pone.0042725)

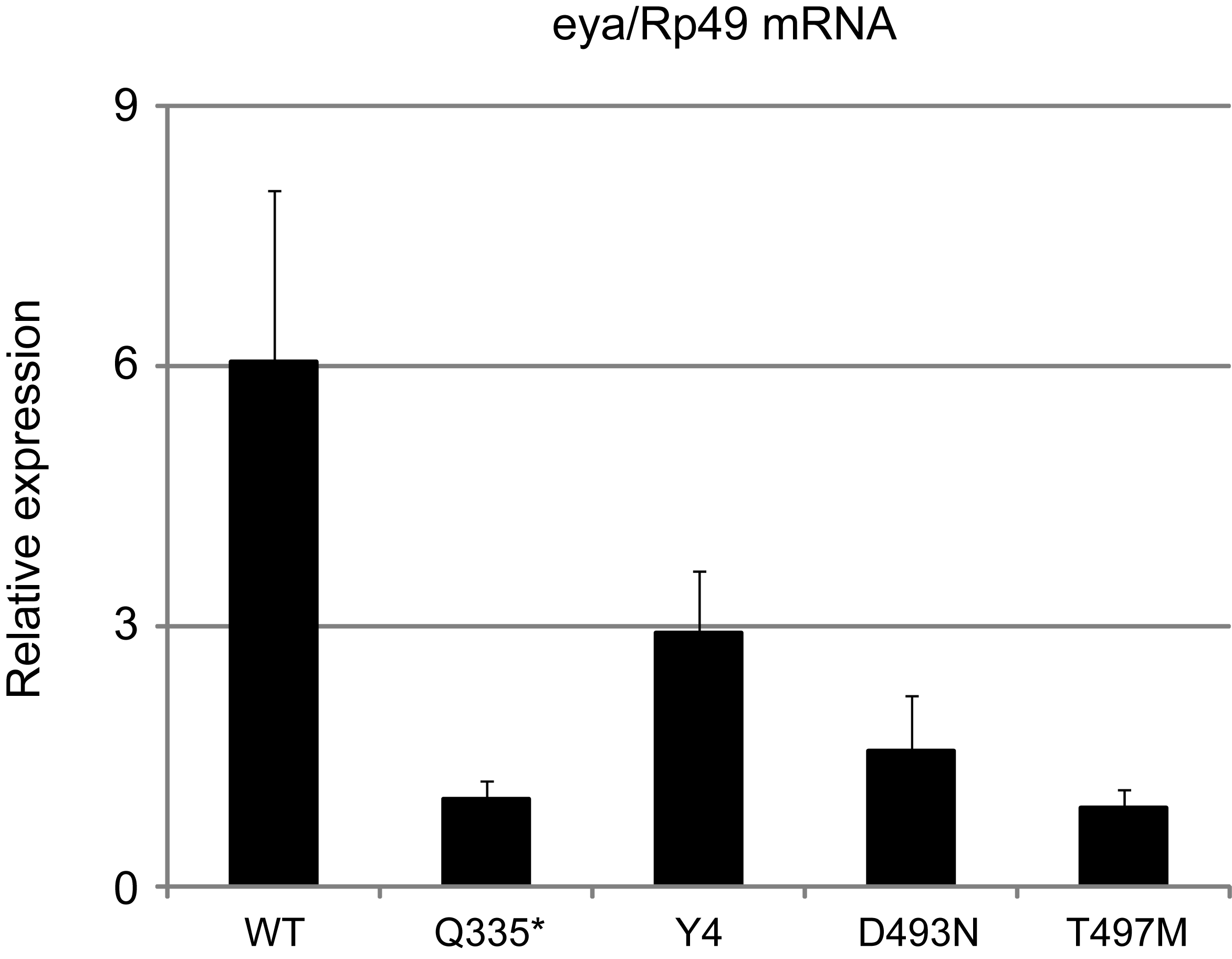

Supplement: Figure S1 — EYA expression in transgenic rescue fly lines. The levels of eya mRNA were measured in rescue transgenic fly lines by quantitative RT-PCR and normalized to Rp49 mRNA levels. The relative expression values to Q355* are indicated. The value represents the average and standard deviation of three independent experiments. A pool of 5–7 adult flies per genotype was collected in each experiment. (TIF) [file pone.0042725.s001.tif]

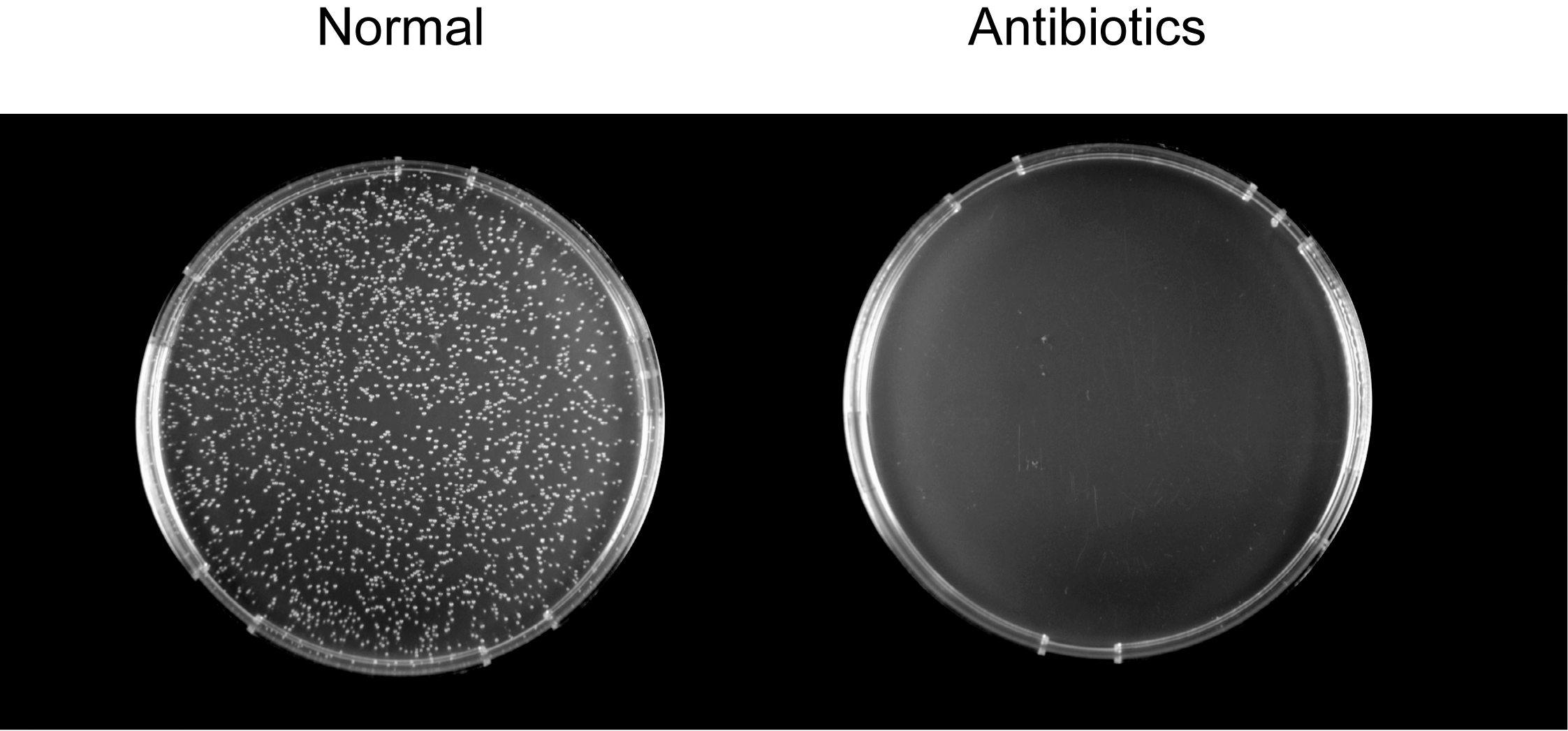

Supplement: Figure S2 — Bacteria growth in Antibiotics-treated flies. Three flies grown in normal or antibiotics medium were squashed in 100 µl of PBS and then spread on LB plates. The LB plates were incubated at 25°C for two days. (TIF) [file pone.0042725.s002.tif]
