# Supplementary material for: Drosophila EYA Regulates the Immune Response against DNA through an Evolutionarily Conserved Threonine Phosphatase Motif
Source: PLoS One. 2012 Aug 15;7(8):e42725. doi: 10.1371/journal.pone.0042725 (PMC3419738; doi:10.1371/journal.pone.0042725)
Supplement: Table S1 — Primers used in this study. (DOCX) [file pone.0042725.s003.docx]

**Table S1**

Primers used in this study

| Name | Direction | Sequence (5’ → 3’) |
| --- | --- | --- |
| eya-RB-N | Fwd | ttgtataatgtgccgtgctatcaaaacttc |
| eya-N | Rev | ttactatcataagaagcccatgtcgaggg |
| N-IKKbeta_F | Fwd | GGGGacaagtttgtacaaaaaagcaggctTCATCACTGTGGTGTTTTGCTTTTCCG |
| N-IKKbeta_R | Rev | GGGGaccactttgtacaagaaagctgggtCTTATCACTAATAGTGATCAATTTTCATTTCATTA |
| N-Rel_F | Fwd | GGGGacaagtttgtacaaaaaagcaggctTCAATCAGTACTACGACCTGGACAATG |
| N-Rel_R | Rev | GGGGaccactttgtacaagaaagctgggtCTTATCACTAAGTTGGGTTAACCAGTAGGGCGTAAG |
| eya Q335* | Fwd | tacaacaacttcgggtagcaggactacggcg |
| eya Q335* | Rev | cgccgtagtcctgctacccgaagttgttgta |
| eya Y4-1 | Fwd | gcggctactacaacgagcaggccggcaacgctgccagtccggccaactactcac |
| eya Y4-1 | Rev | gtgagtagttggccggactggcagcgttgccggcctgctcgttgtagtagccgc |
| eya Y4-2 | Fwd | ttacagtccggccaacgcctcaccgtatgcggtc |
| eya Y4-2 | Rev | gaccgcatacggtgaggcgttggccggactgtaa |
| eya D493N | Fwd | gggtgttcgtctggaatctggacgagacg |
| eya D493N | Rev | cgtctcgtccagattccagacgaacaccc |
| eya T497M | Fwd | gtctgggatctggacgagatgctcatcatcttc |
| eya T497M | Rev | gaagatgatgagcatctcgtccagatcccagac |
| Q-eya | Fwd | actccggatacggaactcct |
| Q-eya | Rev | acagctgcgagttgttgttg |
| Q-Rp49 | Fwd | gacgcttcaagggacagtatctg |
| Q-Rp49 | Rev | aaacgcggttctgcatgag |
| Q-AttA_F | Fwd | ggcccatgccaatttattca |
| Q-AttA_R | Rev | agcaaagaccttggcatcca |
